# What we know and what should we know about the future of blockchain in finance

**DOI:** 10.12688/f1000research.153215.1

**Published:** 2024-09-12

**Authors:** Shikta Singh, Rachana Jaiswal, Shashank Gupta, Chinmoy Kumar

**Affiliations:** 1 School of Management, Kalinga Institute of Industrial Technology, Bhubaneswar, Odisha, 751024, India; 2 Department of Business Management, HNB Garhwal (A Central) University, Srinagar, Srinagar, Uttarakhand, 249161, India; 3 Department of Non-Financial Risk Tech, Morgan Stanley Advantage India Pvt. Ltd, Bengaluru, Karnataka, 560103, India; 4 School of Management, Kalinga Institute of Industrial Technology, Bhubaneswar, Odisha, 751024, India

**Keywords:** Blockchain; finance; VOS viewer; networking analysis; literature review.

## Abstract

**Background:**

In response to the transformative impact of blockchain technology on economic and financial landscapes, there is a critical need for a review study that analyses the knowledge landscape from diverse perspectives.

**Methods:**

This research VOSviewer, and Bibliometrix to undertake a bibliometric analysis of the expanding literature related to blockchain technology within the financial sector. Through a examination of 500 published articles, the study identifies insightful trends, patterns, and emerging domains on a global scale.

**Results:**

The findings highlight the advancing trajectory of blockchain research in finance, with a notable concentration of studies originating from the United States and China, both in terms of total publications and citations. Key thematic clusters identified include “smart contracts,” “financial institutions,” “initial coin offerings,” and “big data analytics.” Intersections with financial risk management, digital transformation, and the integration of big data analytics with artificial intelligence and machine learning are particularly noteworthy, marking focal points of exploration.

**Conclusions:**

While affirming the potential of blockchain, the analysis also sheds light on persistent impediments hindering its widespread adoption and utilization. This study not only contributes to the current understanding of blockchain in finance but also serves as a valuable resource for future researchers. It guides systematic reviews by pinpointing prominent journals and influential authors within the dynamic field of blockchain finance, thereby fostering a deeper understanding and facilitating further exploration in this evolving field.

## 1. Introduction

Blockchain technology stands at the forefront of problem-solving across various industries, presenting a revolutionary approach to collaboration. This technology fosters trust and collaboration among individuals, replacing traditional formalities and paperwork with teamwork, creativity, and efficiency. Its potential lies in creating a more secure and truthful environment, with the eventual eradication of fraud and crime being a conceivable outcome ( Malik et al., 2022). In the realm of finance, blockchain is poised to redefine the rules of the game and reshape the industry’s landscape. Leading financial institutions worldwide are spearheading the adoption of blockchain technology with the aim of enhancing efficiency, creating innovative solutions, deriving tangible business benefits, and ultimately revitalizing the financial sector ( Jaiswal et al., 2022a; 2024d) and other sectors ( Jaiswal, 2022, 2023a, 2023b; Prakhar et al., 2024b). Originating as the backbone of the Bitcoin network in response to the 2008 financial crisis, blockchain employs distributed ledger technology to record the creation, ownership, and sale of valuable assets ( Xu et al., 2019). Its unique feature lies in ensuring the privacy of transactions and records while eliminating the need for a trusted third party, such as a bank, by requiring consensus among all network nodes before any transaction can occur.

The financial sector’s attention to ledger technology is justified, given its potential to enhance security and efficiency. Blockchain facilitates more open and secure business networks, standardized operating models, streamlined procedures, reduced costs, and the introduction of innovative products and services ( Jaiswal et al., 2023; Gupta et al., 2022). Notably, digital securities can be issued more rapidly, cost-effectively, and flexibly, catering to the preferences of individual investors and thereby improving investor markets, issuer costs, and counterparty risk ( Patel et al., 2022). Amid the proliferation of blockchain research, academic interest has surged due to its global significance, security implications, and potential economic impact ( Jaiswal, 2023a, 2023b). This investigation aims to provide a clear and systematic overview of blockchain studies, enabling scholars to grasp the unique features and taxonomy of various study fields ( Wang et al., 2021). Despite the increase in publications related to blockchain in finance research over the past half-decade, there is a need for more quantitative and qualitative research, particularly in understanding business models ( Jaiswal & Gupta, 2023b; Holotiuk et al., 2017). The ongoing pandemic emphasizes the urgency of digitalization in relocating businesses, highlighting blockchain technology’s role in simplifying the transfer of flexible and resilient financial products and securities, potentially paving the way for contactless connectivity in the near future. As the current economic downturn prompts businesses to leverage cutting-edge technologies, the study acknowledges the transformative potential of blockchain technology. The ongoing COVID-19 crisis has underscored the importance of technology for success and flourishing, resulting in an upward trend in publications. The impact of blockchain on financial institutions, including reducing money laundering, countering terrorism financing, enhancing transaction security, and improving transparency, cannot be overstated.

To address the paucity of research on blockchain’s potential applications in the financial industry, this study examines the productivity and competitiveness of the market. Through bibliometric techniques and quantitative tools, the study explores the collaboration between authors, countries, and organizations, building upon previous efforts in the field ( Chen et al., 2021; Haghnazar Koochaksaraei et al., 2021). The research questions (RQs) guiding this study are as follows:

***RQ1.** What is the current publication trend in blockchain finance research?*

***RQ2.** Who are the leading contributors in terms of authors, institutions, and countries in blockchain finance research?*

***RQ3.** Which articles and journals have the most significant impact on blockchain finance research?*

***RQ4.** What are the predominant themes, hotspots, and potential future research directions in blockchain finance research?*

In a prior study conducted by Jaiswal et al. (2022a), the focus was on discerning the fundamental characteristics of published documents within blockchain applications. This was part of the banking industry’s initiative to fortify the digital financial infrastructure. Although that research successfully pinpointed essential services utilizing blockchain technology, it fell short in terms of conducting a thematic analysis and addressing future trends. In contrast, the current study offers a more comprehensive approach by providing a detailed thematic analysis. It not only identifies multiple clusters within the finance sector related to blockchain but also establishes future research agendas.

The insights derived from this analysis carry numerous practical applications. For researchers within the financial sector, this review serves as a panoramic overview of the publication trends in blockchain within finance research. It aids in understanding the scientific community’s evolving interest in the topic over time. Moreover, the review is a valuable resource for aspiring authors, helping them identify key literature, including relevant articles and journals, while also pinpointing potential collaborators—be it authors, institutions, or countries—for blockchain finance research. Additionally, for future authors, the review’s identified themes and topics offer a means to distinguish their work from existing blockchain finance literature, emphasizing the uniqueness of their contributions. The identified research directions act as inspiration, encouraging writers to delve into innovative and meaningful projects. Lastly, these perspectives serve as a preview of forthcoming research, offering valuable insights for policymakers and business professionals.

The subsequent sections of the paper are structured as follows: Section 2 details the database and methodology, Section 3 provides results encompassing publication and citation patterns, along with profiles of the most prolific and influential contributors. Section 4 delves into major and emerging themes, presenting potential developments. Finally, Section 5 concludes the paper and outlines limitations of this study.

## 2. Methods

### 2.1 Database & tools

The Dimensions database stands out as one of the largest accessible resources within the academic and research community, offering free access to users. In recent years, it has been extensively utilized across various research fields ( Jaiswal et al., 2022a, 2023, 2024d; Cortés et al., 2022). Its inclusion of the most influential journals and publishing houses across diverse academic disciplines makes the Dimensions database particularly attractive for bibliometric analysis.

For the construction and visualization of bibliometric networks involving journals, researchers, citations, bibliographic coupling, co-citation, or co-authorship, we employ two software applications: VOSviewer ( Van Eck & Waltman, 2010) and RStudio Bibliometrix ( Aria & Cuccurullo, 2017). These tools offer robust capabilities for visualizing and analyzing bibliometric data ( Jaiswal and Gupta, 2023a, 2023b; Prakhar et al., 2024a; Gupta et al., 2024; Jaiswal et al., 2022b). Notably, VOSviewer comes equipped with text mining features, allowing the construction and visualization of co-occurrence networks of keywords extracted from a body of literature ( Jaiswal and Kumar, 2023; Jaiswal et al., 2024a, 2024b, 2024c, 2024d). These applications have gained widespread use in recent bibliometric research studies ( Jaiswal et al., 2022a, 2024a).

Bibliometric Analysis was performed using VOSViewer ( https://www.vosviewer.com/) and RStudio Biblioshiny ( https://www.bibliometrix.org/home/index.php/layout/biblioshiny).

### 2.2 Article search and selection

The first step in retrieving data is identifying the proper collection of blockchain-related articles published in finance research, as outlined in Table 1. An initial search performed over the period 2018-2022 using the search criteria *((“blockchain” OR “blockchain technology” OR “blockchain technologies”) AND (“finance” OR “banking” OR “financial” OR “bank”))* results in 5,561 documents. These are filtered into the fields *“commerce, management and tourism and services”*, *“business and management”*, and *“banking, finance, and investment”*, producing 1,198 publications. When further screened to exclude conference papers, books, chapter summaries, and other non-articles, the final corpus includes 500 articles, which are examined using bibliometric analysis, which has the capacity to manage vast corpora, to reveal fundamental characteristics, identify publication trends, distinguish advanced topics and construct visualizations of thematic evolution. Both retrospective analysis and future research directions are considered ( Ibrahim & Truby, 2021).

**Table 1.** Search criteria and article selection.

Filtering criteria	Exclusion	Inclusion
Selection Criteria		
Search engine: Dimensions		
Search date: 29 December 2022		
Search Criteria: (Include articles "Titles, abstracts, and keywords" only)		
Search period: 2018-2022		
Search term: (("blockchain" OR "blockchain technology" OR "blockchain		5561
technologies") AND ("finance" OR "banking" OR "financial" OR "bank"))		
Subject area: "Commerce, Management and Tourism and Services," "Business and	4363	1198
Management," "Banking, Finance and Investment,"		
Document type: "Articles"	741	509
Article selection		
Filter the erroneous records: Only documents with the valid author(s)	5	504
Language filtration: Include only English-language documents	4	500
Tools: VOSViewer, Bibliometrix R package		

Data can be obtained from the Dimensions AI database or can be directly downloaded from here: FigShare, “Dimensions-Publication-2022-07-09_04-12-30.csv” (Dataset. https://doi.org/10.6084/m9.figshare.26364760.v1).

## 3. Results and Discussion

In this section, we scrutinize the fundamental attributes of 500 published articles, exploring aspects such as research direction, annual publication presentation, co-author citation, country, organization, and authorship. Additionally, we delve into the interconnections between these various dimensions.

### 3.1 Publication output and growth trend

Blockchain technology has emerged as a recent development in the field of finance. Despite the growing number of financial journal publications, it is noteworthy that only one paper from 2014 has specifically delved into the realm of blockchain within finance literature. Consequently, the analysis primarily focuses on post-2018 publications, as this marks the period when blockchain research in finance began gaining momentum in addressing practical, real-world issues.

In 2022, a total of 162 articles were published on the subject, demonstrating a substantial increase in scholarly interest. This trend is consistent with the preceding years, where 2021 saw 158 articles, 2020 had 144, 2019 featured 73, and 2018 recorded 43. The sheer volume of work dedicated to blockchain technology in finance research underscores the escalating interest among researchers, highlighting a growing inclination towards publishing and contributing to the evolving discourse in this domain.

### 3.2 Productive institutions based on total publications and citations

Table 2 compares the top ten affiliations and institutions based on the total number of citations and publications. The Royal Institute of Technology has the most citations (349), and Hong Kong Polytechnic University has the second- most (307), but for total publications, Hong Kong Polytechnic University has the most (7), followed by the University of Luxembourg (6).

**Table 2.** Most productive institutions for blockchain research in finance from 2018 to 2022.

Top Institutions based on Total Publications				Top Institutions based on Total Citations			
TP	Institution	TC	TLS	TC	Institution	TP	TLS
7	Hong Kong Polytechnic University	307	15	349	Royal Institute of Technology	2	10
5	University of Luxembourg	46	3	307	Hong Kong Polytechnic University	7	15
5	Dublin City University	42	17	238	Worcester Polytechnic Institute	2	11
5	Financial University	13	1	209	Stevens Institute of technology	2	8
4	Amity University	49	4	187	Montpellier Business School	2	2
4	University of Waikato	36	9	161	De Montfort University	3	8
4	Middlesex University	33	3	155	Washington University in St. Louis	3	1
3	De Montfort University	161	8	117	ETH Zurich	3	10
3	Washington University in St. Louis	155	1	109	Illinois Institute of technology	2	0
3	ETH Zurich	117	10	103	Lund University	2	0

Note(s): TC = total citations. TP = total publications. TLS = total link strength.

**Figure 1.**
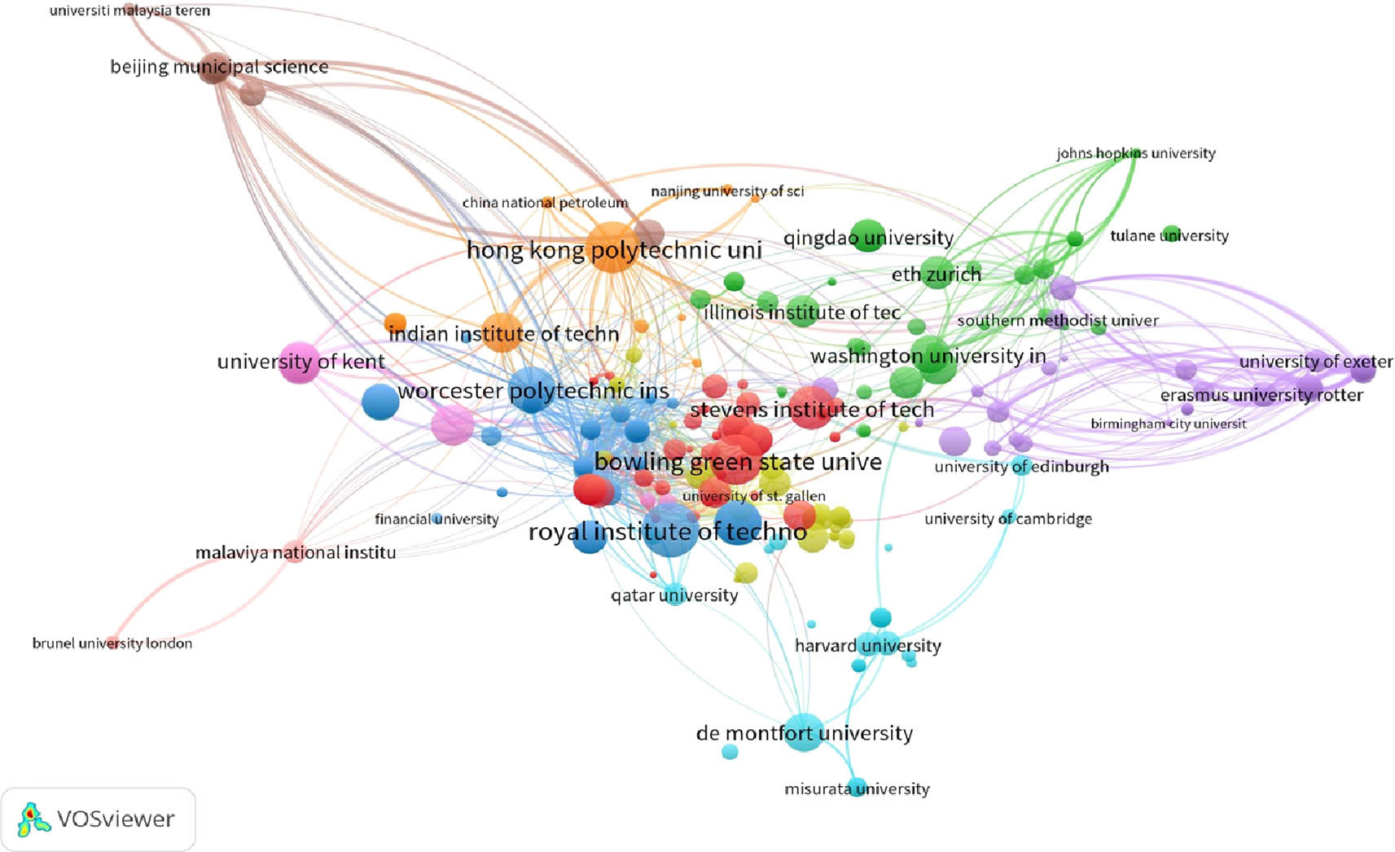
Collaborative networks of publications with institutions.

**Figure 2.**
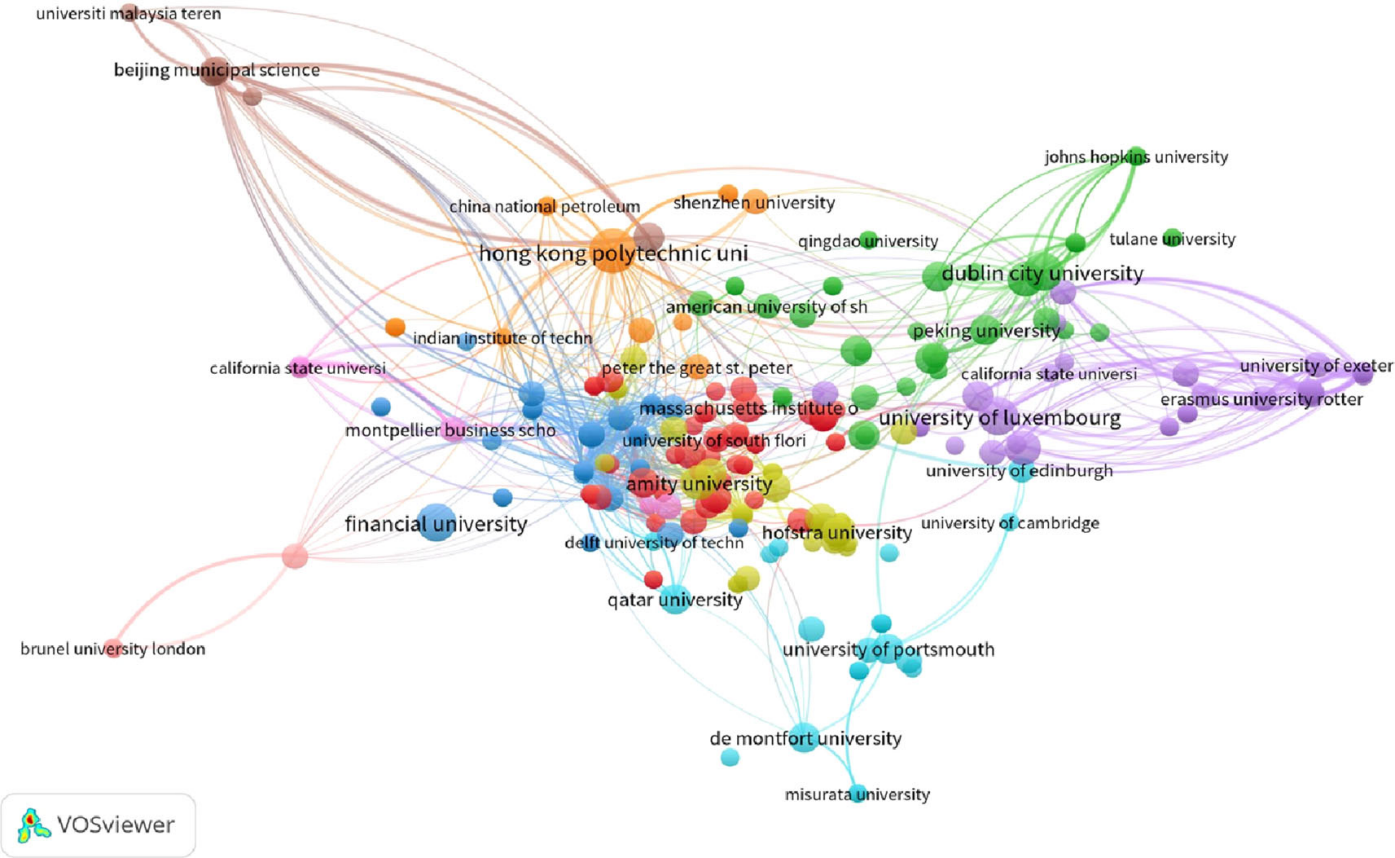
Collaborative networks of citations with institutions.

The VOSviewer user manual specifies that the strength of a link is consistently positive, with a higher value indicating increased reliability. The Total Link Strength (TLS) is determined by the frequency of a specific combination of items (such as keywords, authors, sources, or institutions) appearing in scholarly articles. Figures 1 and 2 illustrate the network visualization of institutions based on their total publications and total citations, respectively. In terms of publications, the Hong Kong Polytechnic University has a Total Link Strength of 15, Dublin City University has a TLS of 17, and ETH Zurich has a TLS of 10. In the context of total citations, the Royal Institute of Technology boasts a TLS of 10, Worcester Polytechnic Institute has a TLS of 11, and Stevens Institute of Technology has a TLS of 8.

### 3.3 Productive journals, countries, and authors in the field of blockchain technology

Table 3 shows that Business Horizons is top of the list for total citations, with 643 citations and 3 publications, followed by Technological Forecasting and Social Change with 512 citations and 14 publications. Notably, the latter journal has a TLS of 40, which is better than any of the journals listed in the top 10.

**Table 3.** Most productive journals for blockchain research in finance from 2018 to 2022.

TC	Sources	TP	TLS
643	Business Horizons	3	25
512	Technological Forecasting and Social Change	14	40
365	Logistics	2	10
336	Journal of Management Information Systems	1	3
251	International Journal of Production Economics	4	18
236	International Journal of Production Research	3	5
179	Transportation Research Part E: Logistics and Transportation Review	1	11
155	Journal of Management Analytics	2	9
148	Journal of Financial Intermediation	1	1
136	Applied Health Economics and Health Policy	1	3

Note(s): TC = total citations. TP = total publications. TLS=total link strength.

**Figure 3.**
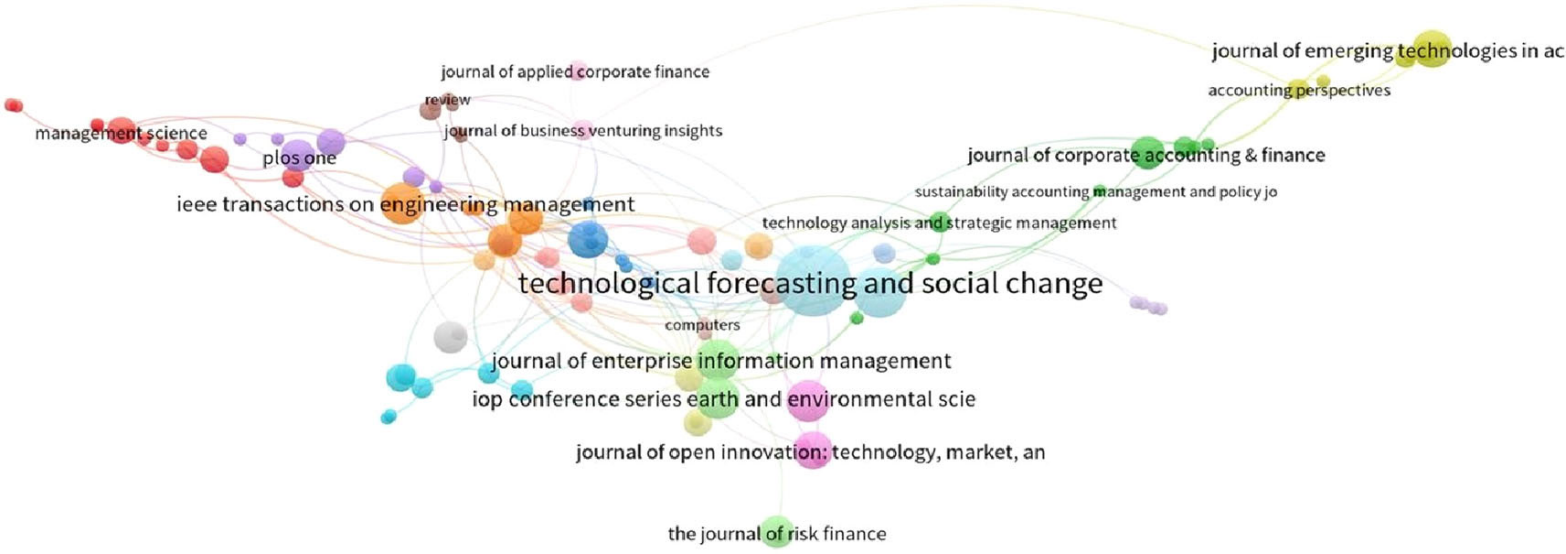
Collaborative networks of productive journals.

Loess, which stands for “locally estimated scatterplot smoothing”, is used for multivariate data. Source dynamics apply loess to the number of documents published from 2018 to 2022 ( Table 4). The Loess procedure is a robust, nonparametric method for smoothing empirical data that contains outliers, offering flexibility, because no assumptions are made regarding the parametric form of the regression surface ( Cleveland and Devlin, 1988). In 2020 and 2021, we observe a sharp increase in the number of publications on blockchain research in finance (see Table 4 and Figure 3). However, as we approach 2022, the loess method moderates this anomaly with a gradual decline.

**Table 4.** Source growth of blockchain research in finance (2018-2022).

Journal Name	2018	2019	2020	2021	2022	TP	TC	Cluster	Link	TLS
Technological Forecasting and Social Change	0	0	6	6	2	14	512	15	27	45
Ieee Transactions on Engineering Management	0	1	4	1	0	6	111	7	6	6
Journal of Enterprise Information Management	0	0	4	2	0	6	124	11	18	22
Managerial Finance	0	0	6	0	0	6	59	9	4	5
International Journal of Recent Technology And Engineering	0	3	2	0	0	5	11	3	1	1
Journal of Emerging Technologies in Accounting	0	3	2	0	0	5	52	4	3	4
Journal of Open Innovation: Technology, Market, and Complexity	0	0	1	4	0	5	38	9	5	5
Annals of Operations Research	0	0	2	1	1	4	94	7	12	14
Business Inform	0	0	0	3	1	4	3	0	0	0
International Journal of Innovative Technology and Exploring Engineering	0	2	1	1	0	4	2	19	1	1
International Journal of Production Economics	0	0	0	3	1	4	251	7	15	20

Note(s): TC = total citations. TP = total publications. TLS=total link strength.

Gilbert (1977) demonstrates that scientists use citations as a persuasive tool. The age of an article is a factor in its citations ( Dang & Li, 2020). According to Schwert (1993), affiliation with the top contributing institutions and nations positively influences citations. Authors affiliated with the most prolific contributing institutions publish more and cite more of their prior work ( Donthu *et al*., 2020). Table 5 indicates that the United States is the leading contributor to blockchain research in finance in terms of total citations and total publications, followed by China for total publications and the United Kingdom for total citations. Although the United Kingdom has produced fewer publications (42) than China (55), it has received slightly more citations (1,101 for the United Kingdom versus 1,082 for China).

**Table 5.** Most productive countries for blockchain research in finance from 2018 to 2022.

Top countries based on total publications				Top countries based on total citations			
TP	Country	TC	TLS	TC	Country	TP	TLS
66	United States	2018	197	2018	United States	66	194
55	China	1082	134	1101	United Kingdom	42	159
42	United Kingdom	1101	165	1082	China	55	132
23	Russia	35	3	503	Sweden	7	26
21	Germany	222	25	387	Australia	10	35
21	India	332	79	370	Switzerland	6	39
16	Italy	128	41	351	France	11	48
11	France	351	50	332	India	21	79
10	Spain	39	9	233	Belgium	5	10
10	Australia	387	35	222	Germany	21	21

Note(s): TC = total citations. TP = total publications. TLS=total link strength.

**Figure 4.**
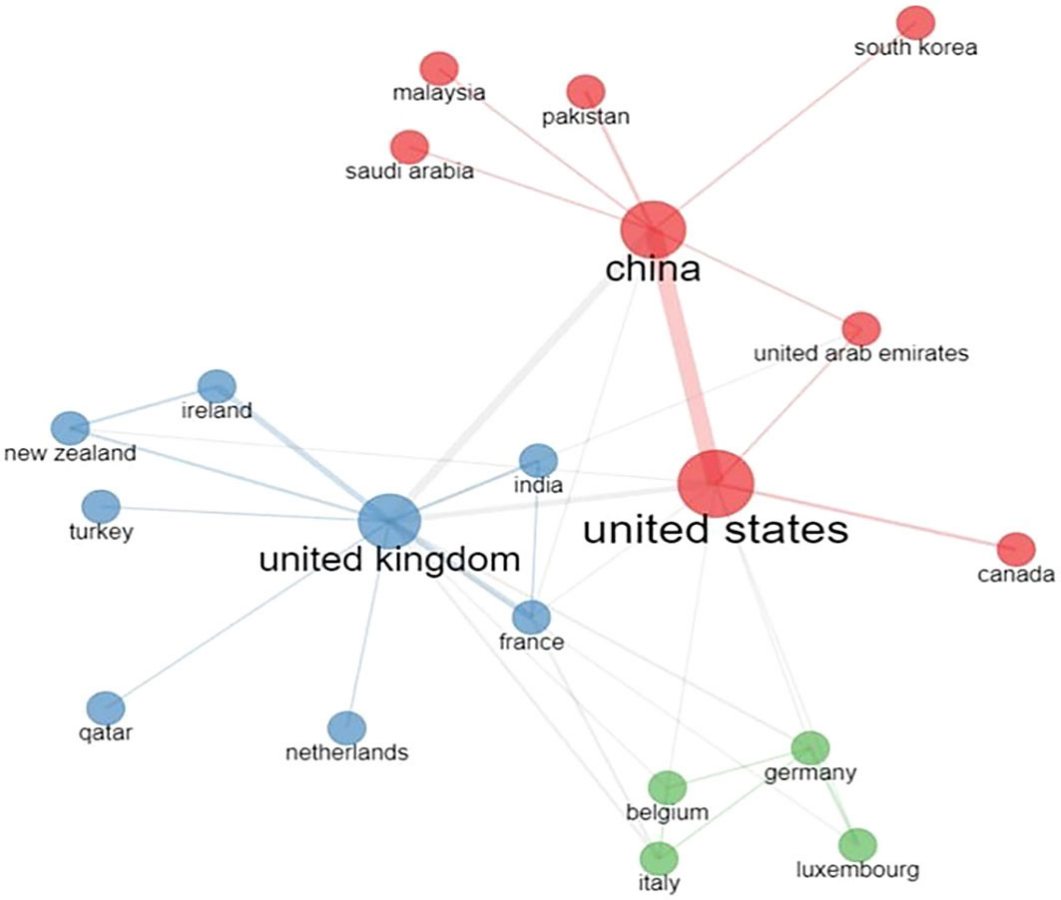
Collaborative networks of countries for blockchain research in finance.

Figure 4 provides insight into clusters of countries. China, the United States, United Arab Emirates, Pakistan, South Korea, Malaysia, and Canada fall into the same cluster, shown in red, while the United Kingdom, India, France, and Turkey belong to the cluster shown in blue. The third cluster, in green, consists of Germany, Italy, Luxembourg, and Belgium.

Lotka (1926) uses a variation of the inverse square law to describe the publication frequency of researchers in a specific field. As a general rule, according to this law, the number of authors who make multiple contributions during a given period is smaller than the number of authors who make just one. The actual number of publications in a given period is compared to the theoretical Lotka curve for the same field to determine an author’s productivity. The author productivity for blockchain research in finance is shown in Table 6, which shows that the blockchain research corpus is dominated by occasional authors with less than five publications, as opposed to core authors who each contribute multiple papers to the body of knowledge. The Lotka curve theory supports this.

**Table 6.** Author productivity based on total publications (2018-2022).

Author	2018	2019	2020	2021	2022	TP	TC
Choi, Tsan-ming	0	0	4	2	0	6	301
Safiullin, MR	0	0	5	0	0	5	3
Stein smith, S.	1	3	0	1	0	5	19
Abdukaeva, A.A.	0	0	4	0	0	4	3
Devi, N.Chitra	0	0	0	1	3	4	1
Kumar, A.	0	1	1	1	1	4	111
Kumari, A.	0	0	0	1	3	4	1
Liu, J.	0	0	1	1	2	4	49
Owen, Robyn	0	3	1	0	0	4	33
Wang, L.	0	0	0	3	1	4	9

Note(s): TC = total citations. TP = total publications

**Figure 5.**
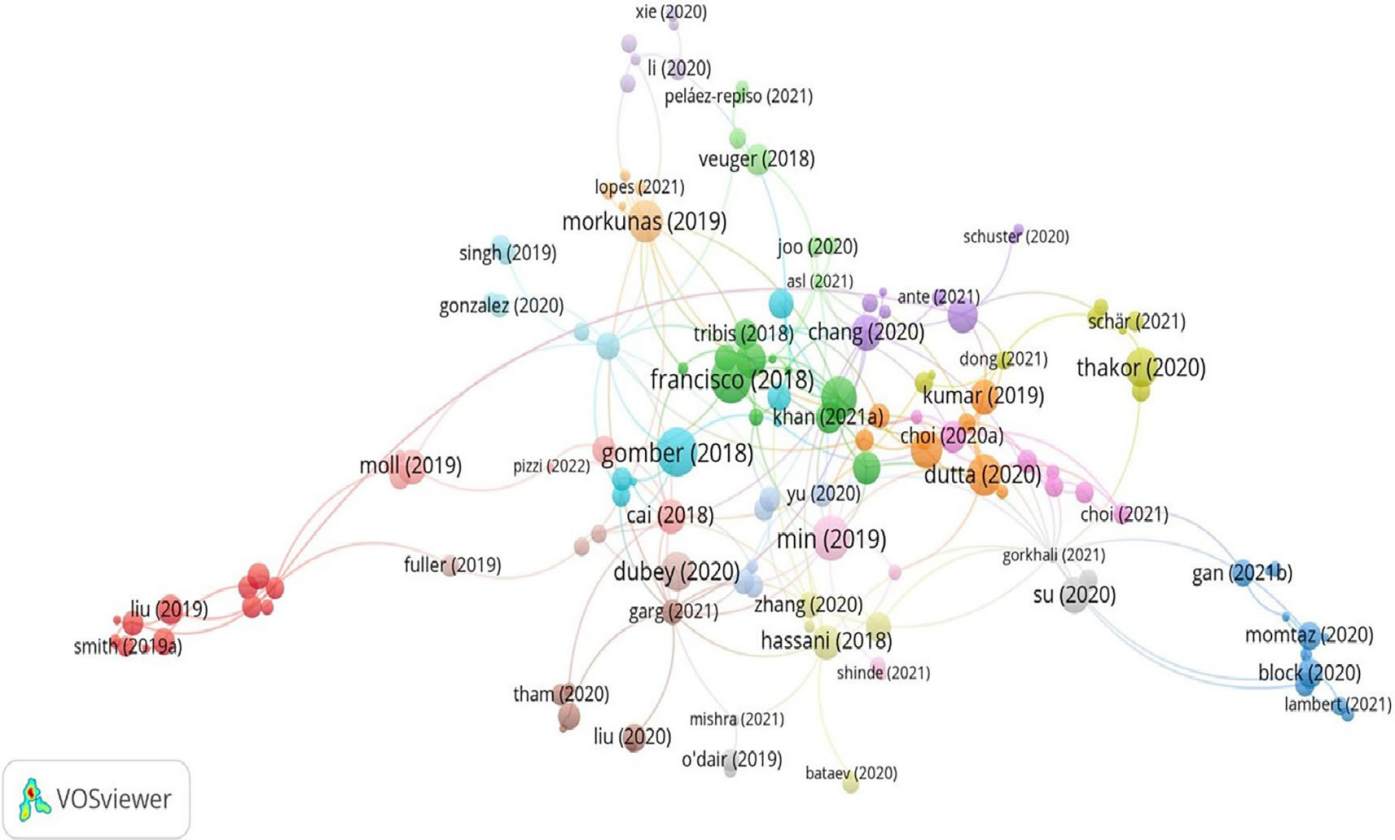
Network collaboration among the top documents.

An author may produce less influential work but cite the work of authors from other countries, thereby indirectly increasing the citation score of locally cited documents in the corpus. Table 7 lists the 15 most-cited articles from 2018 to 2022, while Figure 5 provides the network collaboration among the top documents. Supply chain, the fintech revolution, and financial services are the most frequently cited study areas in the blockchain research in finance ( Moosavi *et al*., 2021). Most of these studies are published by Elsevier, Taylor & Francis, Springer, and MDPI. Logistics, Journal of Management and Information Systems, Business Horizons, International Journal of Production Economics, Journal of Financial Intermediation, Technological Forecasting and Social Change, Journal of Business Venturing, and The Electricity Journal are among the leading journals that publish such articles.

**Table 7.** Most global cited documents for blockchain research in finance for the period 2018-2022.

Paper	Paper Title	Journal name	Publisher	DOI	TC	TCpY	Normalized TC	Cluster	Link
Francisco & Swanson (2018)	The Supply Chain Has No Clothes: Technology Adoption of Blockchain for Supply Chain Transparency	Logistics	MDPI	https://doi.org/10.3390/logistics2010002	359	71.8	10.216	2	7
Gomber *et al.* (2018)	On the fintech revolution: Interpreting the forces of innovation, disruption, and transformation in financial services.	Journal of management information systems	Taylor & Francis	https://doi.org/10.1080/07421222.2018.1440766	336	67.2	9.5619	6	4
Min *et al.* (2016)	Blockchain technology for enhancing supply chain resilience	Business Horizons	Elsevier	https://doi.org/10.1016/j.bushor.2018.08.012	294	73.5	12.051	18	10
Morkunas *et al.* (2019)	How blockchain technologies impact your business model.	Business Horizons	Elsevier	https://doi.org/10.1016/j.bushor.2019.01.009	227	56.8	9.3043	16	14
Kouhizadeh *et al.* (2021)	Blockchain technology and the sustainable supply chain: Theoretically exploring adoption barriers	International journal of production economics	Elsevier	https://doi.org/10.1016/j.ijpe.2020.107831	225	113	46.229	2	10
Dutta *et al.*(2020)	Blockchain technology in supply chain operations: Applications, challenges and research opportunities.	Transportation research part e: Logistics and transportation review	Elsevier	https://doi.org/10.1016/j.tre.2020.102067	179	59.7	12.292	7	10
Dubey *et al.*(2020)	Blockchain technology for enhancing swift-trust, collaboration and resilience within a humanitarian supply chain setting	International journal of Production research	Taylor & Francis	https://doi.org/10.1080/00207543.2020.1722860	166	55.3	11.399	17	3
Thakor(2020)	Fintech and banking: What do we know?.	Journal of financial intermediation	Elsevier	https://doi.org/10.1016/j.jfi.2019.100833	148	49.3	10.163	4	2
Radanović & Likić (2018)	Opportunities for use of blockchain technology in medicine.	Applied health economics and health policy	Springer	https://doi.org/10.1007/s40258-018-0412-8	136	27.2	3.8703	7	3
Angelis & Da Silva (2019)	Blockchain adoption: A value driver perspective	Business Horizons	Elsevier	https://doi.org/10.1016/j.bushor.2018.12.001	122	30.5	5.0006	2	7
Su *et al.*(2020)	Financial implications of fourth industrial revolution: Can bitcoin improve prospects of energy investment?	Technological Forecasting and Social Change	Elsevier	https://doi.org/10.1016/j.techfore.2020.120178	115	38.3	7.897	20	1
Chang *et al.*(2020)	How Blockchain can impact financial services–The overview, challenges and recommendations from expert interviewees.	Technological Forecasting and Social Change	Elsevier	https://doi.org/10.1016/j.techfore.2020.120166	113	37.7	7.7597	5	11
Chen & Bellavitis (2020)	Blockchain disruption and decentralized finance: The rise of decentralized business models	Journal of Business Venturing Insights	Elsevier	https://doi.org/10.1016/j.jbvi.2019.e00151	110	36.7	7.5536	5	7
Li *et al.* (2019)	Blockchain for decentralized transactive energy management system in networked microgrids	The Electricity Journal	Elsevier	https://doi.org/10.1016/j.tej.2019.03.008	103	25.8	4.2218	3	6
Du *et al.*(2019)	Affordances, experimentation and actualization of FinTech: A blockchain implementation study.	The Journal of Strategic Information Systems	Elsevier	https://doi.org/10.1016/j.jsis.2018.10.002	100	25	4.0988	6	1

Note(s): TC = total citations. TCpY = total citations per year.

## 4. Emerging themes and potential developments

### 4.1 Document-level analysis of blockchain research in finance

According to the existing literature, a scientific document’s topicality, methodological rigor, design, core content, and quality should be the primary determinants of citations rather than the individual authors. It is often argued that the contents and quality of an article affect its citations. Valtakoski (2020) refers to scholarly content in a piece of literature, whereas Stremersch *et al*. (2007) argue that longer articles tend to receive more citations. Furthermore, it seems easier to track down an article with more keywords, leading to more citations ( Valtakoski, 2020), whereas it is difficult to search for a lengthy title, which can hurt citations. When analysing the corpus, we consider three elements: trends in the words used, a word tree map, and a word cloud.

**Figure 6.**
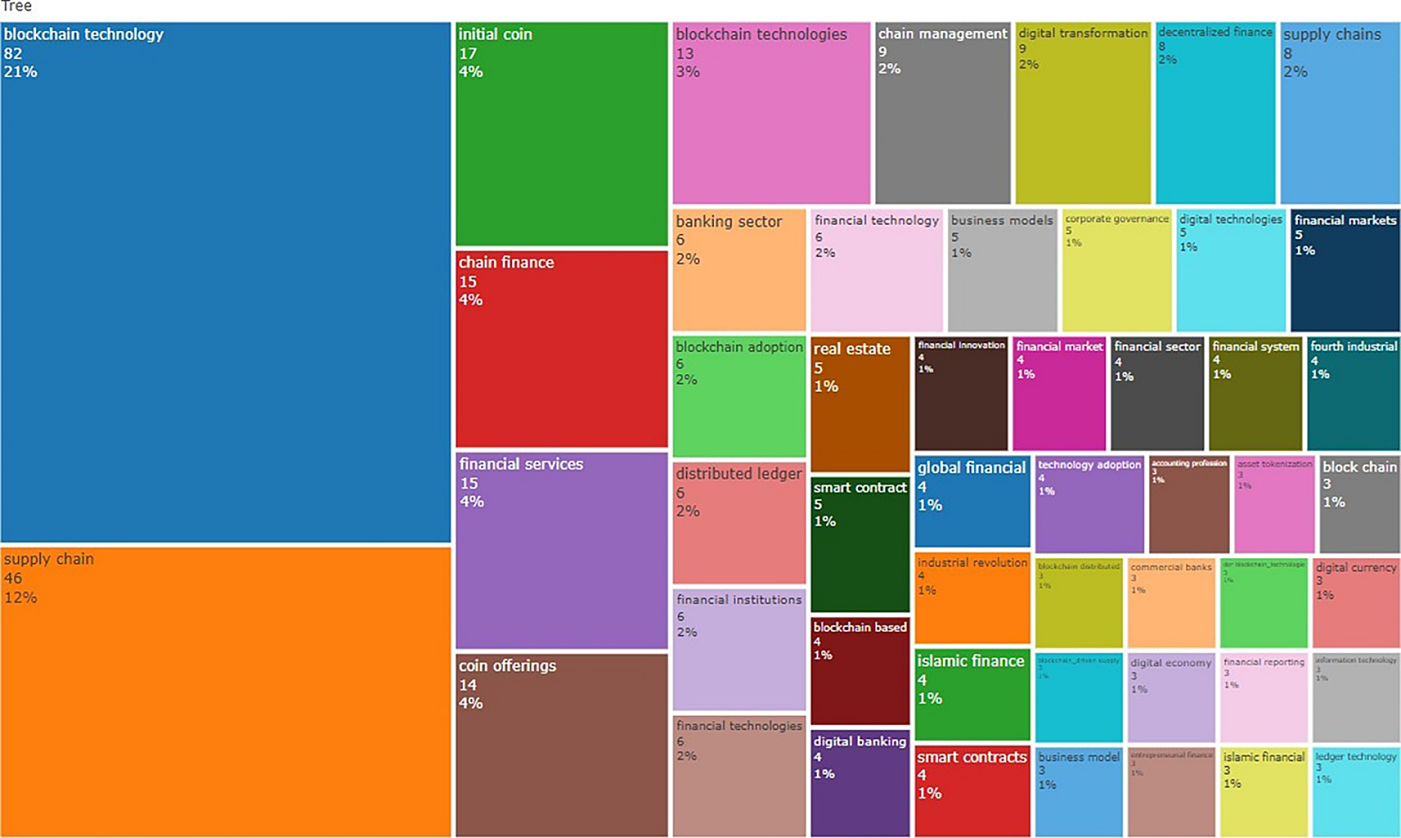
Word tree map based on title frequency using bigrams.

Trending topics based on bigrams applied to the abstracts reveal that Islamic fintech, fintech companies, collaborative innovation, smart contract, and financial innovations are top trending topics for blockchain research in finance. Researchers frequently include a variety of keywords, each of which has the potential to be incorporated into a graphical illustration of the data. The results of applying a tree map to the top fifty keywords in the extracted corpus to construct a proportional map are displayed in Figure 6. The term “blockchain” is by far the most common, but the research is heavily influenced by concepts such as supply chain, initial coin offering (ICO), and financial services.

**Figure 7.**
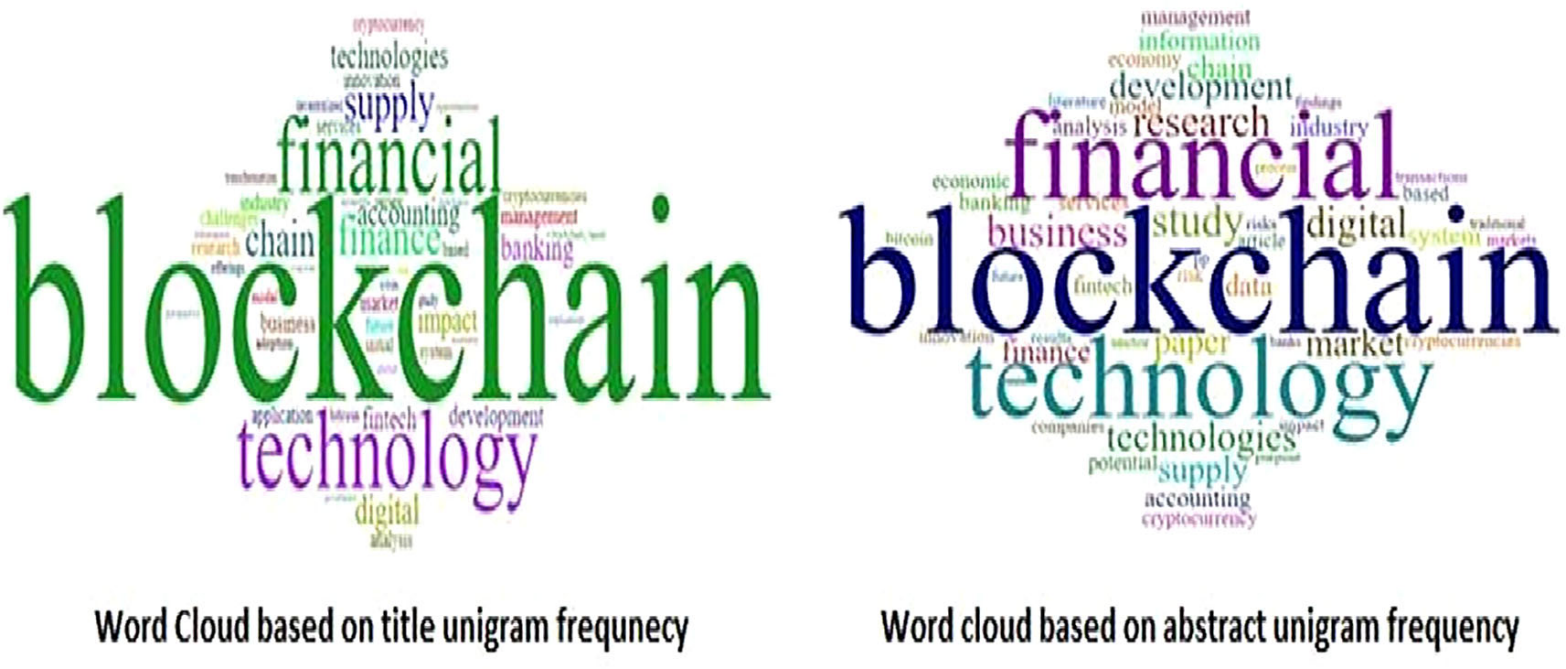
Word cloud based on title and abstract frequency.

The title and abstract unigram frequency distribution of the corpus is used to generate a word cloud (see Figure 7). The word “blockchain” is used most frequently (title 996, abstract 1,514), followed by “financial” (title 108, abstract 1,058), “technology” (title 104, abstract 978), and “finance” (title 104, abstract 978), as highlighted with thick font densities.

### 4.2 Conceptual knowledge structure

To decipher the data contained within a body of accumulated literature, bibliometrics are used, most frequently based on citations. However, new studies having low citations does not necessarily indicate that they are without significance ( Bhukya *et al*., 2022). When new conceptual knowledge structures (CKSs) are researched in the context of their temporal evolution, as cited events, it is much simpler to comprehend the beginnings and development of these structures over time. In order to locate the CKS within this corpus, we make use of a topic dendrogram.

**Figure 8.**
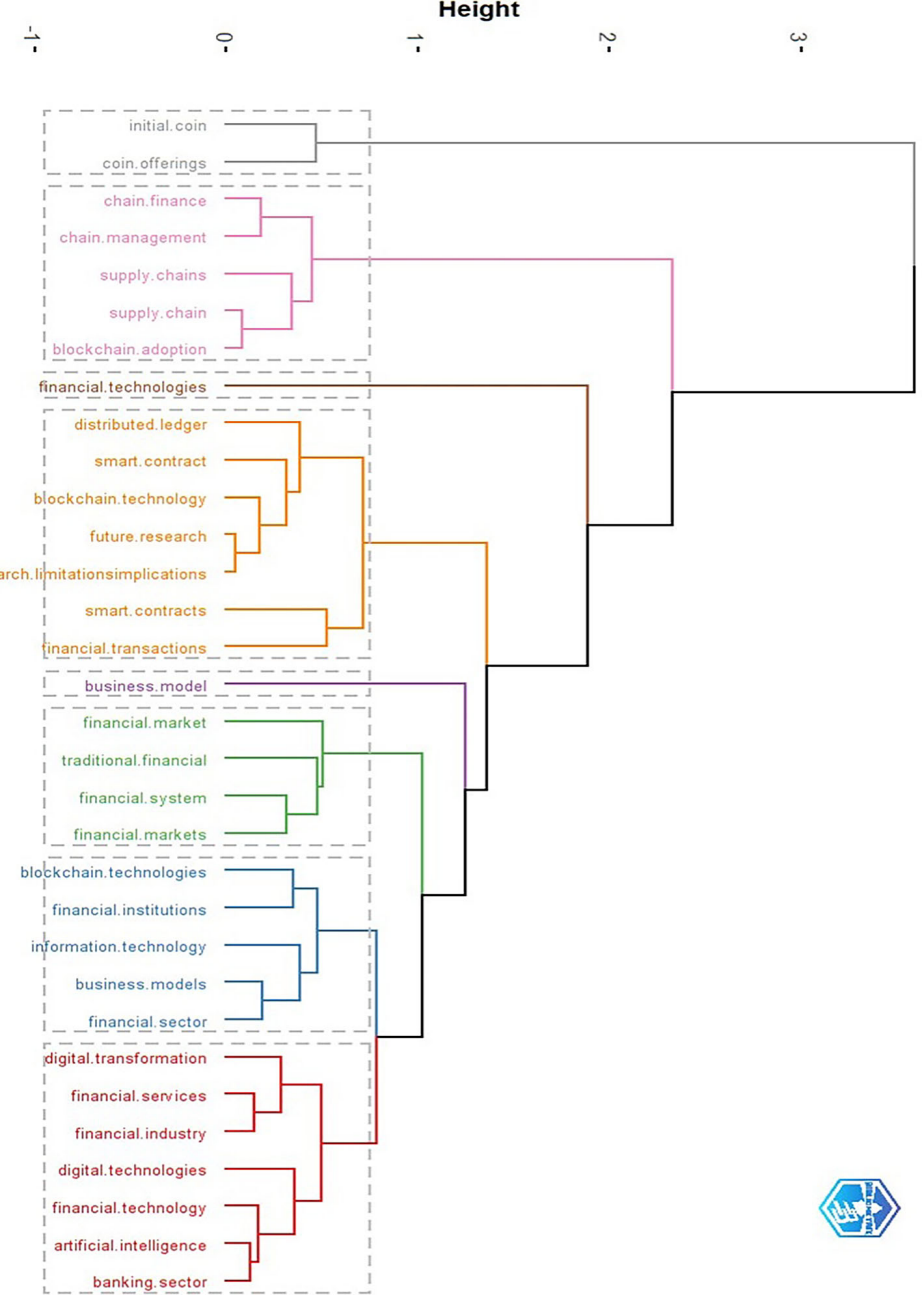
Clustering of blockchain research in finance for topic grouping (Generated using RStudio – Biblioshiny).

This section deals with topic grouping and clustering. We analyse the network diagram with blockchain as the central theme. The surrounding clusters give us an idea of blockchain research in finance. We identify eight distinct clusters with simple manual interpretation and analysis of the dendrogram ( Figure 8). These 8 clusters are labelled ( Table 8) smart contracts, supply chain, financial services, financial institutions, financial market, financial technologies, initial coin offerings, and business model. We provide notable publications with more than 50 citations. It is evident that most of the work is in the area of smart contracts, followed by supply chain, and financial institutions.

**Table 8.** Topic grouping for blockchain research in finance.

Cluster	TA	ATC50
smart contracts	364	Francisco & Swanson (2018); Min (2016); Dutta *et al.* (2020); Dubey *et al.* (2020); Thakor (2020); Radanović & Likić (2018); Angelis & Da Silva (2019); Su *et al.* (2020); Li *et al.* (2019); Du *et al.* (2019); Kumar *et al.* (2020); Hassani *et al.* (2018); Cai (2018); Moll & Yigitbasioglu (2019); Choi (2023); Veuger *et al.* (2018); Khan *et al.* (2021); Tribis *et al.* (2018); Block *et al.* (2021); Liu *et al.* (2019); Demirkan *et al.* (2020); Kouhizadeh *et al.* (2021); Kurpjuweit *et al.* (2021)
supply chain	2	
financial services	4	
financial institutions	70	Gomber *et al.* (2018); Morkunas *et al.* (2019); Chang *et al.* (2020); Chen & Bellavitis (2020)
big data analytics	10	
financial technologies	3	
initial coin offerings	4	
business model	3	

Note(s): TA = total number of articles. ATC50 = articles having total citations greater than 50.

After compiling the content of the existing literature and organizing the data from the clusters, we find that the most active areas of blockchain research are in the following two domains: financial risk management and digital transformation; and big data analytics, coupled with machine learning and artificial intelligence (AI).

4.2.1 Financial risk management and digital transformation

The financial sector pioneered and ultimately made the most effective use of blockchain technology. Many academics study the blockchain for its possible financial uses ( Ali *et al*., 2020; Osmani *et al*., 2020). For example, Cong *et al*. (2021) argue that centralized mining pools for risk sharing would not disrupt decentralized mining based on blockchain technology due to cost settings and energy consumption, providing valuable insight into other consensus protocols and organizations. Distributed ledger technology (DLT) and smart contracts are cited by Pal *et al*. (2021) as the two main aspects of blockchain that can streamline banking and corporate operations. Blockchain technology’s rapid development and updates are set to profoundly impact the nature of businesses and economic and social progress. Blockchain could damage the traditional and centralized business management and organization model because it could lower transaction costs and make it easier to acquire external resources. Treiblmaier (2018) develops a research framework based on four economic theories (principal-agent theory, transaction cost analysis, resource-based view, and network theory) to investigate how blockchain has modified supply chain management.

4.2.2 Big data analytics coupled with artificial intelligence and machine learning

The theory and technology behind machine learning and its potential uses have advanced rapidly since its inception ( Jaiswal and Gupta, 2024; Jaiswal et al., 2023; Jaiswal and Kumar, 2023; Jaiswal and Medhavi, 2018). Much research attention is focused on the intersection of machine learning, AI, and blockchain technology. Tanwar *et al*. (2019) combine machine learning algorithms with blockchain to ensure accurate data analysis results. Outchakoucht *et al*. (2017) propose a distributed and dynamic policy to control access to the internet of things (IoT). Meanwhile, dynamic optimization and self-adjustment of policy are made possible by the combination of machine learning and reinforcement learning algorithms. A blockchain and AI-based architecture for the IoT has been developed by Singh *et al*. (2020), representing a powerful approach to integrating the two technologies. The authors conduct a qualitative and quantitative evaluation of the proposed architecture, showing that it is superior to current IoT architectures.

There are several challenges to the adoption of blockchain in finance, including regulatory challenges ( Guo & Liang, 2016), security and privacy of data ( Deshpande *et al*., 2017), interoperability ( Kshetri, 2018), the energy cost of the technology ( Hassani *et al*., 2018), uncertainty about how to govern technology ( Attaran & Gunasekaran, 2019), and technical and scalability challenges.

### 4.3 Future research avenues

In this section, while briefly discussing the eight thematic clusters, we provide some future research questions (FRQs) based on the content analysis of the articles in each cluster.

4.3.1 Smart contracts

Smart contracts can help with finance in a variety of ways. They facilitate secure and automated transactions, reduce costs and paperwork, streamline processes, and make payments more efficient ( Rozario & Thomas, 2019). Smart contracts can also be used for the digital tokenization of assets such as stocks, bonds, and real estate as well as for automated loan management, escrow services, and trading ( Cai, 2018). Additionally, smart contracts can facilitate payment processing, verify identity, and authenticate documents ( Dubey *et al*., 2020). Future research should explore the following FRQs:

***FRQ1.** How can smart contracts be utilized to increase financial inclusion and accessibility for underbanked populations?*

***FRQ2.** What are the potential risks and limitations of using smart contracts in finance and how can they be mitigated?*

***FRQ3.** How can smart contracts be integrated with existing financial systems and regulations to ensure compliance and stability?*

***FRQ4.** Can smart contracts enhance transparency and accountability in financial transactions and institutions?*

***FRQ5.** How can smart contracts be developed and improved to support more complex financial instruments and transactions?*

4.3.2 Supply chains

Blockchain technology can revolutionize the way supply chain finance is conducted. By using a distributed ledger system, businesses can securely and transparently track the movement of goods and money through the supply chain. This would allow for improved visibility and streamline the process of financial transactions. Blockchain technology could also be used to securely store data related to invoices, payments, and contracts, making it easier to access and update information in real- time. This would help improve efficiency and reduce the costs associated with supply chain finance ( Kurpjuweit *et al*., 2021). Furthermore, blockchain technology can facilitate the emergence of decentralized, peer-to-peer financing models, which could open the door to more efficient and cost- effective financing options ( Kouhizadeh *et al*., 2021; Dubey *et al*., 2020). Future research should explore the following FRQs:

***FRQ6.** How can blockchain technology be used to enhance transparency and trust in supply chain finance?*

***FRQ7.** How can blockchain technology be integrated with existing supply chain systems and technologies to maximize benefits?*

***FRQ8.** What are the potential risks and limitations of using blockchain in supply chain finance, and how can they be addressed?*

***FRQ9.** Can blockchain technology facilitate the development of new, more inclusive, and sustainable financing models in the supply chain?*

***FRQ10.** How can blockchain technology be used to improve the speed and efficiency of supply chain finance transactions?*

4.3.3 Financial services

Blockchain technology can improve many aspects of the financial services industry by providing more transparency and faster, more secure, efficient, and cheaper transactions, including international payments ( Meng & Du, 2021). Blockchain can also reduce the risk of fraud and help combat money laundering ( Chang *et al*., 2020). Additionally, it can provide access to financial services to those who previously did not have access. Blockchain can facilitate the transfer of assets, such as stocks and bonds, and the trading of digital assets, such as cryptocurrencies. Future research should explore the following FRQs:

***FRQ11.** How can blockchain technology be used to increase financial inclusion and accessibility for underbanked populations?*

***FRQ12.** What are the potential risks and limitations of using blockchain in finance, and how can they be mitigated?*

***FRQ13.** How can blockchain technology be integrated with existing financial systems and regulations to ensure compliance and stability?*

***FRQ14.** Can blockchain technology enhance transparency and accountability in financial transactions and institutions?*

***FRQ15.** How can blockchain technology be developed and improved to support more complex financial instruments and transactions?*

4.3.4 Financial institutions

Blockchain technology has the potential to provide financial institutions with a variety of benefits, including improved security, lower costs, faster transactions, and greater transparency ( Sun *et al*., 2021). With blockchain, financial institutions can increase the security of their transactions by using distributed ledgers and smart contracts that are cryptographically secure. This technology can also reduce the costs associated with verifying transactions, since a network of computers automatically validates them. Blockchain transactions are faster than traditional methods, as they are conducted directly between two parties in a matter of minutes, compared to days or weeks with traditional methods ( Basori & Ariffin, 2022). Notably, blockchain technology can increase transparency by providing an immutable record of all transactions that can be accessed by all parties, allowing for greater trust in the system. Future research should explore the following FRQs:

***FRQ16.** How can blockchain technology be used to improve the efficiency and scalability of financial institutions?*

***FRQ17.** What are the potential risks and limitations of using blockchain in the financial industry, and how can they be addressed?*

***FRQ18.** How can blockchain technology be integrated with existing financial systems and regulations to ensure compliance and stability?*

***FRQ19.** Can blockchain technology help financial institutions to provide a higher level of customer service and satisfaction?*

***FRQ20.** How can blockchain technology be used to increase transparency and accountability in financial transactions and institutions?*

4.3.5 Financial market

Blockchain technology has the potential to revolutionize the financial market. It can be used to store and share data, facilitate transactions, and provide access to financial services for individuals and businesses. Blockchain technology can also improve transparency and security in the financial market by providing a secure, distributed ledger that records all transactions. This can help reduce the risk of fraud and cyber threats ( Somin *et al*., 2020). Blockchain technology can also be used to develop new products and services, such as smart contracts and decentralized exchanges, that benefit investors and other market participants ( Gorkhali & Chowdhury, 2022). Future research should explore the following FRQs:

***FRQ21.** How can blockchain technology be used to improve the accessibility and affordability of financial services for individuals and businesses?*

***FRQ22.** What are the potential risks and limitations of using blockchain in the financial market, and how can they be mitigated?*

***FRQ23.** How can blockchain technology be integrated with existing financial systems and regulations to ensure compliance and stability?*

***FRQ24.** Can blockchain technology enhance the transparency and accountability of financial transactions and institutions?*

***FRQ25.** How can blockchain technology be developed and improved to support more complex financial instruments and transactions?*

4.3.6 Financial technologies

Blockchain technology has the potential to revolutionize the financial services industry by providing a secure, efficient, and cost-effective way to move money, store data, and facilitate transactions between parties ( Bataev *et al*., 2020). Blockchain allows for high levels of transparency and trust as all records are immutable, meaning they cannot be changed or altered in any way. This technology can also offer greater security for financial transactions as all records are stored on a distributed ledger that is viewable by all participants. Blockchain can also streamline processes such as digital payments, smart contracts, and more. By leveraging blockchain, financial institutions can reduce costs, increase efficiency, and open up new business opportunities. It can revolutionize financial services by enabling transactions to be processed more quickly and securely, with higher transparency and traceability ( Sun *et al*., 2022). Companies are already using blockchain technology to improve the efficiency of payments, transfers, and other financial processes. Blockchain can also be used to facilitate faster and more secure access to financial data, as well as to provide more efficient recordkeeping for financial institutions. Additionally, blockchain could be used to build digital currencies, such as cryptocurrencies, that could be used for payments and other financial activities ( Brus, 2020). Future research should explore the following FRQs:

***FRQ26.** What are blockchain technology’s specific applications and use cases in the financial services industry?*

***FRQ27.** How can blockchain technology be integrated into existing financial systems and processes?*

***FRQ28.** What are the potential challenges and barriers to adopting blockchain technology in the financial services industry?*

***FRQ29.** How can blockchain technology be used to enhance the security and privacy of financial transactions?*

***FRQ30.** What are the benefits and drawbacks of decentralized financial systems enabled by blockchain technology?*

***FRQ31.** How can blockchain technology be used to improve access to financial services for underbanked populations?*

***FRQ32.** What are blockchain technology’s regulatory and legal implications in the financial services industry?*

4.3.7 Initial coin offerings

Blockchain technology can be used for initial coin offering (ICO) fundraising. ICOs are a form of crowdfunding that involves the sale of digital tokens, usually based on blockchain technology. Using blockchain technology, ICOs can provide investors with a more secure, transparent, and efficient way to participate in fundraising. This can be done by providing a platform for project creators to issue digital tokens and for investors to purchase them using cryptocurrency ( Momtaz, 2021). Once the tokens are issued, the proceeds from the sale can be used for project development, marketing, and other related expenses. Additionally, blockchain technology can provide an immutable ledger to track the ownership of the tokens and facilitate the transfer of tokens between investors ( Colombo *et al*., 2022). Future research should explore the following FRQs:

***FRQ33.** How effective are ICOs compared to traditional forms of fundraising, such as equity and debt financing?*

***FRQ34.** What impact does the use of blockchain technology have on the security and transparency of ICOs?*

***FRQ35.** How can blockchain be used to improve the regulatory compliance of ICOs and protect investors from fraud?*

***FRQ36.** What role do smart contracts play in the ICO process, and how can they be used to automate and streamline the process?*

***FRQ37.** How can the use of ICOs and blockchain technology change the fundraising landscape for startups and emerging businesses?*

4.3.8 Business models

Blockchain technology has become a significant focus of business-minded individuals due to its potential to revolutionize business operations. The technology can create new business models and transform existing ones ( Ioannou & Demirel, 2022). For example, it can create a secure and transparent transaction environment and provide a shared data record across multiple organizations ( Suwanposri *et al*., 2021). It also has the potential to automate processes such as payments, contracts, and other transactions. Using blockchain technology, businesses can benefit from cost savings, improved efficiency, and enhanced trust and security. Additionally, the technology can create new business models based on a distributed ledger, allowing multiple parties to participate in transactions without needing a centralized authority ( Baba *et al*., 2021; Khan *et al*., 2021). Future research should explore the following FRQs:

***FRQ38.** How can businesses implement blockchain technology to realize its full potential for transforming operations?*

***FRQ39.** What are the challenges and limitations of using blockchain technology in business, and how can they be addressed?*

***FRQ40.** How can blockchain technology be used to create new business models and revenue streams for businesses?*

***FRQ41.** What is the impact of blockchain technology on existing business models, such as supply chain management, and how can it be optimized?*

***FRQ42.** How can blockchain technology increase trust, security, and transparency in business transactions?*

## 5. Conclusion

This paper combines science mapping and bibliometric analysis to explore the antecedents of blockchain technology research in the finance domain using the Dimensions database. It draws on the top productive journals, countries, institutions, authors, and network collaborations to identify linkages in the blockchain technology field and reveal the foundational themes of blockchain research. The results show a growing trend in research publications for blockchain technology and its application in the financial sector, particularly in the United States, China, and the United Kingdom, which are the most productive and cited countries. There is a growing trend for blockchain research among journals, and Technological Forecasting and Social Change is the most productive journal in this field with the highest number of links, total link strength, and second highest number of citations after Business Horizons. The study highlights the top- cited documents and hence the most influential titles.

A word tree map and word cloud show the focus areas of blockchain technology and its applications in finance. The results indicate that blockchain technology has expanded into supply chains, ICOs, and financial services, and has the potential to develop broader applications in asset tokenization, cryptocurrencies, and decentralized finance. Dendrograms are used to cluster topics and their current statuses, identifying two hotspot trends: financial risk management and digital transformation; and big data analytics coupled with AI and machine learning. While blockchain has proved capable of making the payment process more efficient, with faster transaction settlements and financial institutions saving on international transactions, this study finds multiple roadblocks to the use of blockchain technology in finance, such as regulation, interoperability, energy costs, uncertainty about how to govern it, and technical and scalability problems.

Future research could address some of the limitations of this study, although it provides several valuable insights into blockchain technology in finance. Future studies could apply the same methodology but focus on other industries harnessing a single dataset (Scopus, IEEE, or Web of Science) or a hybrid dataset (a combination of Scopus, Dimensions, Web of Science, etc.). There is room for further investigation into blockchain’s role in the reinvention of other financing, such as trade finance, letters of credit, and invoice factoring. This study uncovers several avenues for future study. The financial sector is rife with regulations, but banks and other financial institutions are beginning to see the potential of blockchain and digital currencies. Exploring how businesses use blockchain-based solutions for open, accessible, and trustworthy financial transactions will be fascinating, as the major players in these sectors experiment to uncover novel use cases and opportunities. In addition, researchers may want to zero in on how blockchain has enhanced transaction processing and interoperability to demonstrate its value to the financial sector.

### Ethics and consent

This study did not involve human participants or animals, and thus did not require informed consent. The analysis was conducted using publicly available data from published articles in the Dimensions database. All sources of data are properly cited, and no personal or sensitive information was used. The study adheres to the ethical standards of research integrity and academic honesty. All data sources are publicly accessible and properly cited.

## Data Availability

Data is available and can be downloaded from here: FigShare, https://doi.org/10.6084/m9.figshare.26364760.v1 ( Jaiswal, 2024). This project contains the following underlying data:
1. Dimensions-Publication-2022-07-09_04-12-30.csv Data are available under the terms of the Creative Commons Attribution 4.0 International license (CC-BY 4.0). This study followed the PRISMA (Preferred Reporting Items for Systematic Reviews and Meta-Analyses) guidelines where applicable to ensure comprehensive and transparent reporting of the bibliometric analysis. Detailed steps were taken to systematically search, select, and analyze the literature using robust analytical tools such as the Dimensions database, VOSviewer, and Bibliometrix. Please see PRISMA Checklist for article “What we know and what should we know about the future of blockchain in finance”. figshare. Figure. https://doi.org/10.6084/m9.figshare.26805244.v1
