# Supplementary material for: What we know and what should we know about the future of blockchain in finance
Source: F1000Res. 2024 Sep 12;13:1051. [Version 1] doi: 10.12688/f1000research.153215.1 (PMC12163367; doi:10.12688/f1000research.153215.1)
Supplement: Peer review history

# Reviewer report: Reviewer response for version 1

**DOI:** 10.5256/f1000research.168078.r329387

**Published:** 2025-06-12

**Authors:** Tapas Mishra

**Affiliations:** 1 University of Southampton, Southampton, England, UK

Blockchain is an inevitable element of growth in finance, enabling security features of operations and empowering reliable interconnectivity of financial units. I appreciate the authors' insights by undertaking a scientific approach to literature review on the subject. The methodology is correct and the results presented are insightful. Perhaps, I would advise the authors to minimise on the number of research questions (including the future research questions... there are too many... in one case, I find this to be more than 34!). There has to be a balance between the broad research questions (that should include many sub-categories) and the theoretical foundation.

Is the work clearly and accurately presented and does it cite the current literature?

Yes

If applicable, is the statistical analysis and its interpretation appropriate?

Yes

Are all the source data underlying the results available to ensure full reproducibility?

Yes

Is the study design appropriate and is the work technically sound?

Yes

Are the conclusions drawn adequately supported by the results?

Yes

Are sufficient details of methods and analysis provided to allow replication by others?

Yes

Reviewer Expertise:

Cryptocurrency, digital finance, methodology

I confirm that I have read this submission and believe that I have an appropriate level of expertise to confirm that it is of an acceptable scientific standard.

# Reviewer report: Reviewer response for version 1

**DOI:** 10.5256/f1000research.168078.r325958

**Published:** 2024-10-17

**Authors:** Nitin Liladhar Rane

**Affiliations:** 1 University of Mumbai, Mumbai, Maharashtra, India

"What we know and what should we know about the future of blockchain in finance" Authors' have made a good attempt by highlighting the advancing trajectory of blockchain research in finance, with a notable concentration of studies originating from the United States and China, both in terms of total publications and citations. Key thematic clusters identified include “smart contracts,” “financial institutions,” “initial coin offerings,” and “big data analytics.” Intersections with financial risk management, digital transformation, and the integration of big data analytics with artificial intelligence and machine learning are particularly noteworthy, marking focal points of exploration.

Is the work clearly and accurately presented and does it cite the current literature?

Yes

If applicable, is the statistical analysis and its interpretation appropriate?

Yes

Are all the source data underlying the results available to ensure full reproducibility?

Yes

Is the study design appropriate and is the work technically sound?

Yes

Are the conclusions drawn adequately supported by the results?

Yes

Are sufficient details of methods and analysis provided to allow replication by others?

Yes

Reviewer Expertise:

AI

I confirm that I have read this submission and believe that I have an appropriate level of expertise to confirm that it is of an acceptable scientific standard.
